# Dysphagia in the intensive care unit—Screening requirements

**DOI:** 10.1111/aas.70035

**Published:** 2025-04-22

**Authors:** Joerg C. Schefold, Daniela Bertschi, Philipp Venetz, Bodil Steen Rasmussen

**Affiliations:** ^1^ Department of Intensive Care Medicine Inselspital, Bern University Hospital, University of Bern Bern Switzerland; ^2^ Department of Anaesthesia and Intensive Care Aalborg University Hospital Aalborg Denmark

**Keywords:** critical illness, deglutition disorder, ICU‐acquired swallowing dysfunction, mortality, post‐extubation dysphagia, sepsis, swallowing dysfunction

Dysphagia, that is, the disability to swallow safely, is present in a considerable number of ICU patients post‐critical illness.^1^ Large‐scale prospective studies with systematic screening indicate that about 18% of ICU patients admitted for emergency reasons and invasive mechanically ventilated are affected by dysphagia post‐extubation.^2^ Despite the fact that dysphagia was shown an independent predictor of increased short‐ and long‐term mortality in both “neurological” and “non‐neurological” (i.e., mixed medical‐surgical) ICU patients,^2^, ^3^ dysphagia still seems an under‐recognized and somewhat overlooked medical condition. Importantly, ICU patients are often discharged from ICUs with unrecognized dysphagia, since many ICUs have not installed systematic routine dysphagia screening following extubation or decannulation.^4^

In this issue of *Acta Anasthesesiologica Scandinavia*, Schandl et al.^5^ validate the *Swedish* version of the Gugging Swallowing Screen—Intensive Care Unit (GUSS‐IVA) score. Following screening of 56 included ICU patients with prolonged intubation (defined as intubation/mechanical ventilation for >48 h), GUSS‐IVA was compared to the diagnostic gold standard of dysphagia, that is, the instrumental exam of fiber‐optic evaluation of swallowing (FEES). In the patient cohort under investigation, the incidence of dysphagia was high (82.4%), which might be somewhat related to the fact that informed consent was required by individuals participating in the study. Importantly, high sensitivity and specificity of GUSS‐IVA to detect dysphagia were observed (area under the curve 0.85, 95% confidence interval 0.71–0.95, positive predictive value 97%). For tracheostomized patients, both a sensitivity and specificity of 100% were noted. After comparison to the gold standard, the authors find that GUSS‐IVA, that is, the Swedish version of GUSS‐ICU, is a valid screening tool to identify ICU patients with dysphagia.

The work of Schandl et al. appears important in several ways. First, their work paves the way for a broad national introduction of dysphagia assessment on ICUs in *Sweden*, potentially also combined with a FEES‐training program for ICU physicians. Second, even though different dysphagia assessment programs are currently proposed for critically ill patients,^6^ the dysphagia assessment algorithm investigated by Schandl et al. should be considered a valid alternative. Finally, however, it may be more important to install a dysphagia assessment program on a given ICU, rather than which exact dysphagia assessment program is applied. Local ICU management should determine which dysphagia assessment program best suits local needs, and especially on large‐volume ICUs, a most pragmatic approach could be chosen. In our personal experience, it appears important to note that dysphagia screening can be performed by trained ICU nurses (Step 1), with dysphagia specialists only assigned to cases of screening positivity (Step 2, preferably complemented by a FEES examination by a trained ICU physician). If installed properly, such a two‐step focused dysphagia assessment program might even save rather than spend ICU staff resources.

The data presented by Schandl et al.^5^ add to mounting data that dysphagia is indeed a major medical problem on ICUs, in particular following prolonged mechanical ventilation.^7^ The presented data might further underline the need for systematic dysphagia assessment in all ICU patients, regardless of the etiology of critical illness.

## FUNDING INFORMATION

None.

## CONFLICT OF INTEREST STATEMENT

The Department of Intensive Care Medicine has research and development/consulting contracts (full disclosure) with Orion Corporation, Abbott Nutrition International, B. Braun Medical AG, CSEM SA, Edwards Lifesciences Services GmbH/SA, Kenta Biotech Ltd, Maquet Critical Care AB, Omnicare Clinical Research AG, Nestlé, Cytel, and Phagenesis Ltd. No personal financial gain resulted from respective development/consulting contracts and/or grants.
